# Use of a Time-of-Flight Camera With an Omek Beckon™ Framework to Analyze, Evaluate and Correct in Real Time the Verticality of Multiple Sclerosis Patients during Exercise

**DOI:** 10.3390/ijerph10115807

**Published:** 2013-11-04

**Authors:** Gonzalo Eguíluz, María Begoña García

**Affiliations:** DeustoTech-Life Unit, DeustoTech Institute of Technology, University of Deusto, Bilbao 48007, Spain; E-Mail: mbgarciazapi@deusto.es

**Keywords:** multiple sclerosis, time-of-flight, verticality improvement

## Abstract

Any person with Multiple Sclerosis (MS), regardless of the severity of their disability, needs regular physical activity. Poorly performed exercises could aggravate muscle imbalances and worsen the patient’s health. In this paper, we propose a human body verticality detection system using a time-of-flight camera as a tool to detect incorrect postures and improve them in real time. The prototype uses Omek’s Beckon™ Framework to analyze and evaluate the position of patients during exercise. Preliminary results, based on objective questionnaires, indicate an improvement in patients’ evolution through better positions and performance of the exercises.

## 1. Introduction

Multiple sclerosis (MS) is a chronic inflammatory demyelinating disease (CIDD) of the central nervous system leading to progressive impairment of various Central Nervous System (CNS) [1] that poses many complications for the affected person; both physical and psychosocial. The latter are the most disruptive as regards the patient’s psycho-emotional balance on certain occasions [2,3,4,5,6]. 

There is currently no medical nor rehabilitative action effective against MS [7,8,9]. Patients should clearly understand that they have to live with certain limitations, and they must adapt physically and psychologically to such limitations [10]. Patients with chronic disorders need to exercise and keep exercising continuously to improve their condition and motor skills. Some people will be able to undertake their rehabilitation at home by themselves with occasional contact from a health professional. Others will need more intensive care and support. The treatments the patients must undergo, such as physiotherapy, medical, psychological and occupational therapy would not return them to their previous situation, but would help to relieve the symptoms, to delay or avoid the progress of the disease, and try to make their quality of life as acceptable as possible. At the same time, the family also has to adjust to this new situation and understand the key role they play in the patient’s treatment. 

Each MS patient has a unique set of symptoms and circumstances that requires a personalized combination of rehabilitation techniques [11]. Evaluation is the first step. In addition to assessing patients’ physical abilities, cognition, and personal goals, evaluation may also include an assessment of their environment to see if modifications to their home or workplace would be useful [12]. 

In addition to the complexity associated with the highly variable course of the disease and its multiple forms, patients with MS experience a wide range of symptoms classified as primary, secondary and tertiary [13]:

- **Primary symptoms** are a direct result of demyelination. This hinders the transmission of electric signals to the muscles (not allowing them to move properly) and body organs (not allowing them to perform normal functions). Many of these symptoms can be managed effectively with medication, rehabilitation, and other medical treatments [13]. The most important symptoms include spasticity (spasticity is stiff or rigid muscles; it may also be called unusual “tightness” or increased muscle tone; reflexes—for example, a knee-jerk reflex—are stronger or exaggerated; the condition can interfere with walking, movement, or speech), weakness, tremor, imbalance, numbness and pain.
- **Secondary symptoms** result from or are consequences of the primary symptoms. Paralysis (a primary symptom) can lead to pressure sores and urinary incontinence can cause recurrent urinary tract infections.
- **Tertiary symptoms** are the social, vocational and psychological complications of the primary and secondary symptoms. The most important symptoms include social, professional, marital psychological problems. Depression, for example, is a common problem among people with MS.

Rehabilitation is a comprehensive and continuous time-limited process with defined goals to promote and achieve optimal levels of physical independence and functional abilities of people with disabilities. It also envisages achieving the necessary psychological, social, vocational and economic levels so that they can lead an independent life. Rehabilitation is a complex process that results from the integration of many procedures to ensure that patients regain a better status, both at home and in the community. Types of care in rehabilitation are shown below [14,15,16]:

- **Institutions-Based Rehabilitation (IBR)**. In an institutions-based approach, all or almost all rehabilitation services are provided by the institution. These services are organized according to those which are available although they no longer correspond to real needs.
- **Institutional rehabilitation based on community outreach**. With outreach, the focus of control is still institution-based. More people can be “reached” but there will be limits according to distance from the institution, and whether the needs of the disabled people are similar to what the institution offers.
- **Community-Based Rehabilitation (CBR)**. This approach covers all the situations in which rehabilitation resources are available within the community. CBR is generated in the same community. It is based on the needs of the person and seeks to solve problems rather than apply techniques or exercises from the health profession.

Rehabilitation based on video and multimedia applications, either web-based or stand-alone applications, tries to make the therapeutic process more attractive to the patient, increasing motivation and improving treatment efficacy [17,18,19,20,21,22]. These approaches incorporate an environment where the patient is able to work with an interactive application to carry out the rehabilitation in an innovative way. The rehabilitation-associated improvements may also be capable of detecting incorrect movements and provide sensorial and/or visual feedback to the patient and/or physiotherapist. 

In this project, we intend to address the following points:

- To re-educate and maintain all available voluntary control.
- To maintain the whole amplitude of the movement of joints and soft tissues, and to teach the patient and/or the relatives adequate tightening procedures to prevent contractures.
- To make treatment techniques a part of everyday life, relating them with appropriate daily activities, ensuring maintenance of all the improvement obtained in this manner.
- To analyze and evaluate in real-time the position of patients during the workout session to avoid unsatisfactory practices that may result in more severe muscle imbalances and worsen their health.

## 2. Related Work

Rehabilitation at home refers to tele-health systems that enable patients to carry out the rehabilitation exercises when it suits them with professional supervision. Tele-rehabilitation applications are effective, as concluded in [23,24,25]. Examples of these applications are described below:

- The **Home Care Activity Desk (HCAD)** [26] project was sponsored by the EC during the period 2003–2005. It dealt with the development of a tele-rehabilitation system to enable patients affected by MS, Stroke (S) or Traumatic Brain Injury (TBI) to perform upper limb rehabilitation treatment at home. An activity desk was purposely designed to allow the patient to perform exercises at home, to monitor patient’s performances, and to transmit the monitored data to a hospital environment. Patents also had the possibility of interacting with the therapist through a teleconference system.
- **eRehab (ubiquitous multidevice personalised telerehabilitation platform)** [27]. This project aims to develop and validate the eRehab platform, a tele-rehabilitation platform based on personalized health service massive deployment architecture. This platform will make it possible to carry out therapies in different environments, such as hospitals, homes and on the go, using the device, user interface and contents that best suit the needs and preferences of each user.
- **AXARM (Extensible Remote Assistance and Monitoring Tool for Tele-Rehabilitation)** [28]. This project is a videoconference oriented and enhanced system which allows specialized professionals from a rehabilitation center, such as psychologists, neurologists and rehabilitators, to carry out remote rehabilitation sessions. Patients can remain at home using broadband communications and Internet services.
- **HELLODOC (Healthcare sErvice Linking teLerehabilitatiOn to Disabled people and Clinicians)** [29]. The main objective of the HELLODOC project was the evaluation of the EU market in terms of home care services including MS, TBI and stroke though a home-based rehabilitation platform [30]. Remote monitoring and control is possible with two webcams and a teleconferencing service. The main parameters of the exercises (*i.e.*, duration, success, number of attempts) are sent electronically and on video tape to the hospital.

The study of literature shows that specific tools for MS are very restrictive focusing on only one rehabilitation treatment (ours includes six mayor areas of rehabilitation in MS: speech therapy, neuropsychology, occupational therapy, yoga, physiotherapy and rehabilitation) and none includes a tracking system for physical rehabilitation to analyze and evaluate the position during the exercise.

## 3. Methods

### 3.1. Participants

The Basque Multiple Sclerosis Foundation (ADEMBI) selected five of its patients whose rehabilitation focused on the upper-body and all of whom used wheelchairs; and two of its professionals. All of the patients showed a low dependency level. Therefore, they did not need help during the pilot test. The five patients were men, whose age ranged between 29 and 40. The group comprised English teachers (*n* = 2) and technical-operators (*n* = 3). As regards professionals, both were women and physiotherapists. Their ages were 25 and 32, respectively. 

### 3.2. Materials

ADEMBI created a set of exercises and questions to carry out the pilot test. These materials included pictures, both static and animated (*n* = 307, 162 MB), videos (*n* = 201, 1.45 GB) and documents (*n* = 104, 21 MB), which are classified according to six main categories: speech therapy, neuropsychology, occupational therapy, yoga, physiotherapy and rehabilitation. Videos and images are used to teach the patients in how to perform their rehabilitation through exercises (*n* = 439) developed by ADEMBI. All of this material was included in a web application, developed *ad-hoc* and connected with a tracking system based on DS311 cameras. The web application relies on Joomla (a Content Management System or CMS), and every module was developed with web technologies like PHP (a widely-used Open Source general-purpose scripting language that is especially suited for web development), JavaScript (a dynamic scripting language supporting prototype based object construction) and Ajax (a web development technique for creating RIAs running on the client’s browser, maintaining communication asynchronously with the server in the background).

Along with this, we developed a system which tracked the movement of patients and detected their position, in particular, the verticality of the patients’ trunk with a time-of-flight (ToF) camera. ToF cameras are active sensors that measure distances based on the phase-shift principle [31]. Camera LEDs emit a light beam in a periodic sinusoidal waveform, in the infrared spectrum, to make it possible to distinguish it from the background light. This signal acts on the objects in the scene and bounces back to the CCD or CMOS sensor of the camera, which will have experienced a delay depending on the distance to the objects that are in the form of reflected signals. The exact time of arrival is calculated for every pixel of the sensor matrix. Each pixel may demodulate the signal, and through its phase, the distance is detected. With the time between transmission and reception of this pulse, the distance is detected by comparing the phases of the light emitted and reflected, generating a gray scale image with depth information [32].

**Figure 1:**
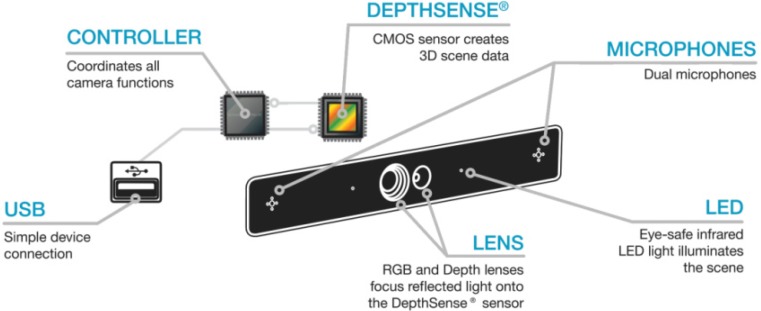
DS311 internals and technology.

In order to achieve this, we used the Beckon™ framework developed by Omek Interactive, Ltd. [33], along with a ToF camera, specifically the Depthsense^®^ 311 (DS311) camera created by SoftKinetic, Inc. [34], see Figure 1.

DS311 internals consist of an OPT8130 ToF sensor with one pair of single-ended outputs, a VSP5324 which acts as an Analog Front End (AFE) and an OPT9110, which acts as a Time of Flight Controller (TFC), all of them manufactured by Texas Instruments (Dallas, TX, USA) [35], (see Figure 1). Table 1 shows the main characteristics of Depthsense^®^ 311 (DS311).

**Table 1:** Depthsense® 311 (DS311) main characteristics.

Item	Value
Depth field of view	57.3° × 42° × 73.8° (H × V × D)
Depth resolution	QQVGA
Frame rate	25–60 fps
Nominal operating range	15 cm–1 m & 1.5 m–4.5 m
Depth noise	<3 cm @ 3 m
Illumination type	LED
RGB resolution	VGA
RGB field of view	50° × 40° × 60° (H × V × D)

Nowadays, there are several frameworks that offer detecting and tracking functionality based on the users’ skeleton. The following table (see Table 2) lists these frameworks:

**Table 2:** Available tracking frameworks.

SDK	Company	Price	Works with DS311 camera?
Kinect SDK 1.7	Microsoft	Free	No
OpenNI SDK 2.2.0.30	PrimeSense	Free	No
NiTE 2.2.0.10	PrimeSense	Free	No
IISU Pro 3.6	Softkinetic	1,200 €	Yes
Beckon SDK 3.0	Omek	Free (Not available *)	Yes
Intel Perceptual Computing R5	Intel	Free	No

* Intel buys Israeli startup Omek Interactive for close to $50 million [36].

As seen from Table 2, the only frameworks that work with DS311 camera are developed by Softkinetic™ (licensed based) and Omek™ (free). The rest of the frameworks work with cameras based on structured light, such as Microsoft™ and PrimeSense™ cameras, while the framework offered by Intel^©^ works only with the Senz3D™ ToF camera developed by Creative^®^. This camera, Senz3D™, is based on the Softkinetic’s DS325 camera, which is focused on hand tracking (short range). In our case, a ToF camera provides better results compared to Kinect™ and other structured light cameras in terms of:

- **Tracking seated people**. Kinect™ sensor offers two tracking modes, default and seated [37]. Seated mode only tracks ten upper-body joints (shoulders, elbows, wrists, arms and head). In our developed system, we needed a spine joint to obtain the trunk’s verticality angle. In version 2.0 of OpenNI^®^ framework, upper body tracking functionality has been deleted because of errors and limitations [38]. Meanwhile, with Beckon™ SDK, the user can select individual joints to track, *i.e.*, upper body plus spine/hips joints.

To finalize this section, detailed information of Beckon™ framework will be found in Section 4, Beckon™ SDK.

### 3.3. Procedure

A professional met patients individually at the ADEMBI center in a session that lasted 20 min on average to explain the aim of the pilot test and the duration. The following week, both professional and patient had another session, one hour on average, to explain everything about the tool developed. During one week, patients tested the system and then, for four weeks, professionals assigned exercises, eight exercises on average, and patients performed them. After this period of time, two satisfaction surveys, one focused on the benefits of the system (Table 3), and another focused on the usability of the system (Table 4) were filled out by the patients. Questionnaire no. 1, benefits to patients, was a 10 item questionnaire with four response options; with values from 1 to 4: a value of 1 represents totally disagree (TD) and 4 represents totally agree (TA) (Table 3). Questionnaire no. 2, usability of the system, was a 10 item questionnaire with five response options; with values from 0 to 4: a value of 0 represents totally disagree and 4 represents totally agree (Table 4). This questionnaire follows the guidelines of System Usability Scale (SUS) [39]. SUS is a reliable tool for measuring usability. It consists of a 10 item questionnaire with five response options for respondents; from strongly agree to strongly disagree. It allows evaluation of a wide variety of products and services, including hardware, software, mobile devices, websites and applications.

**Table 3:** Questionnaire no. 1: Benefits to patients.

#	Question	Responses			
		TD			TA
1	Before using the tool, I think that my health problems were worse than other people in the same situation	1	2	3	4
2	I think that my health has improved by using this system in comparison to other systems	1	2	3	4
3	When I use the system for the time set by the professional, I think that my health improves	1	2	3	4
4	I agree with the frequency of use of the system established by the professional	1	2	3	4
5	I think that I would like to use the system frequently, because it helps me to improve my quality of life	1	2	3	4
6	I believe that my health has improved compared to people who have not used the system	1	2	3	4
7	After using the system, I think that I am more independent (dressing, toileting, *etc.*)	1	2	3	4
8	Have you felt fatigued after each session using the system?	1	2	3	4
9	I had cramps and/or muscle stiffness after each session using the system	1	2	3	4
10	I felt pain or discomfort after each session using the system	1	2	3	4

TD: Totally Disagree; TA: Totally Agree.

**Table 4:** Questionnaire no. 2: Usability of the system.

#	Question	Responses				
		TD				TA
1	The system includes demonstrations that allowed me to observe and practice complex processes new to me	1	2	3	4	5
2	I think that the system interface clearly displays information, is easy to understand and consistent	1	2	3	4	5
3	I felt comfortable and confident using the system	1	2	3	4	5
4	I think that the system modules were consistent and do their job properly	1	2	3	4	5
5	I knew what I was doing at all times	1	2	3	4	5
6	I was able to perform all actions of the system	1	2	3	4	5
7	I was able to read every option of the system	1	2	3	4	5
8	I knew why I was doing the processes at all times	1	2	3	4	5
9	I found the various functions in this system to be well integrated	1	2	3	4	5
10	I needed to learn a lot of things before I could get going with this system	1	2	3	4	5

TD: Totally Disagree; TA: Totally Agree.

## 4. Beckon™ SDK

Beckon™ supports all major 3D depth sensors, allowing to develop products using the best sensor for each application, and to adapt applications developed to take advantage of new sensors as they are introduced. The Beckon™ framework comes with a set of predefined gestures which can be used to design most device and application interfaces.

**Figure 2:**
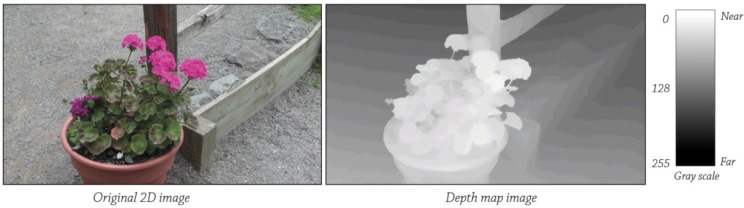
Depth Map.

Beckon™ framework takes the data provided by a depth sensor, a depth map, and analyzes it for information about the scene being viewed. A depth map (see Figure 2) has pixel values corresponding to the distance. For example, brighter values are equal to the shortest distance, or vice versa. The processes used in calculating these parameters are the estimation for describing the intensity of the curves, movement patterns, surface normal and curvatures, *etc.* An intensity image has more than 250,000 pixels and each of them stores 8 bits for the gray level and 8 bits for the color vector components. Pixels also store x, y, z values, which are the coordinates calculated by the scene sensors [40].

Using computer vision techniques, Beckon™ framework identifies the humans in the scene and separates them from the scene background. This step is carried out by image segmentation. The literature covers image segmentation in four main groups [41,42,43]: (a) Methods based on pixels, local (based on properties of pixels and its surroundings) and global (based on global information obtained, *i.e.* the histogram of the image; (b) Methods based on edges; (c) Methods based on regions, using the notions of homogeneity and geometric proximity as growth techniques, merger or division; and (d) Model-based/ knowledge-based segmentation methods. There are some works [44,45] in the literature that use depth images to subtract the background from an image. These works are benefitting from the depth image obtained by the camera to accelerate the background segmentation process.

**Figure 3:**
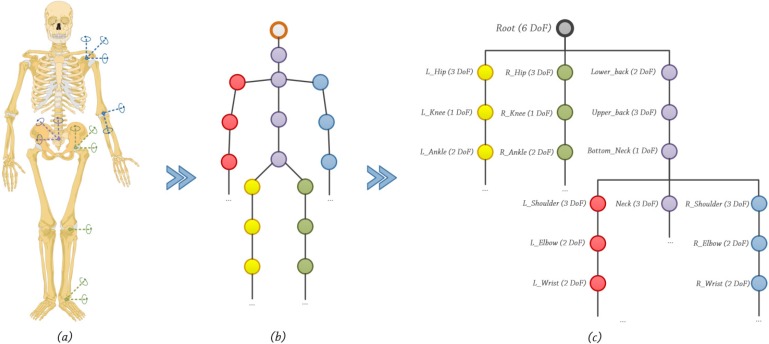
(**a**) Human skeleton. (**b**) Skeletal joints. (**c**) Skeletal joints graph.

Once the image is segmented, Beckon™ applies an initial basic skeletal framework to each human body in the scene, and then enhances that skeleton with a full inverse kinematic skeleton model. Inverse kinematics (IK) is usually considered to be a tool for the animation of skeletons. Before defining what IK is, it is necessary to ﬁrst specify what is meant by skeleton. A skeleton is an articulated structure consisting of bones connected by revolving joints, see Figure 3b. All bones are perfectly rigid in length *l_i_* . Every *i-th* bone has an associated coordinate systems 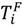 and 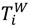. 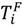 is a coordinate system defined according to a previous bone in structure. 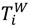 is a coordinate system in the World Coordinate System (WCS). Under these conditions, the following equation is valid, Equation (1):

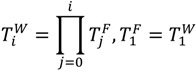
(1)


In practice, the sub-graph, see Figure 3c, is constructed to speed up the traversal and to reduce computations only to those bones which are possibly affected by motion [46,47]. A posture is simply a skeletal configuration. However, not all postures are acceptable. In order to be realistic, they must satisfy a set of criteria. For instance, the natural limits of the articulations should not be violated (physical laws should also be taken into account), and inter-penetration of the body with other objects or with itself is not permitted. 

There are a variety of possible joint types, see Figure 3a. Each joint provides a local rotation and each bone a local translation with different Degrees of Freedom (DoF). Rather than specifying the value of each individual degree of freedom, the IK method automatically computes these values in order to satisfy a given task usually expressed in a Cartesian coordinate system (a Cartesian coordinate system specifies each point uniquely in a plane by a pair of numerical coordinates, which are the signed distances from the point to two fixed perpendicular directed lines, measured in the same unit of length) via estimating each individual degree of freedom. IK techniques provide direct control over the placement of an end-effector object at the end of a kinematic chain of joints, solving the joint rotations which place the object at the desired location [48,49].

## 5. System Design

The system is divided into two main parts: web application with several functionalities and the correction system. In this paper we will address the part of the correction system with the DS311 camera. In summary, the web application part uses Joomla which acts as the infrastructure for data and modules handling. Joomla was chosen because it offers modularity, plenty of plug-ins, and a moderate learning curve. The data storage is powered by MySQL. Finally, the system runs in a Linux environment, specifically the Ubuntu Server environment, with Apache server, an Open Source HTTP Server. We selected these technologies in order to create an accessible, powerful, low-cost and easy-to-use tool.

### Correction System

The correction system analyzes and corrects in real time the verticality of the patient’s trunk (positions leaning forward or leaning backwards not included). It is a desktop application, due to limitations of the Adobe^®^ Flash^®^ wrapper offered by Beckon™. It needs to be executed by the user (or a support person) before the exercise starts. 

**Figure 4:**
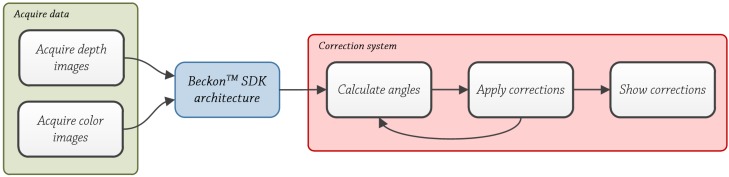
Correction system block diagram.

It consists of three main blocks (see Figure 4): Acquire data, Beckon™ SDK and Correction system. First two, Acquire depth images and Beckon™ SDK will be explained briefly and the third one, the correction system will be explained in detail. 

#### Acquire Data

This block is performed automatically by the camera, the DS311 model. Acquiring color images and depth images are performed simultaneously by the camera through two lenses, one for acquiring color images and one for acquiring depth images. 

To obtain color images, DS311 camera uses an Active Pixel Sensor (APS) based on CMOS technology. A CMOS image sensor is a chip that converts light to electrical signals, and it is made in a complementary metal oxide semiconductor process [50]. It includes amplifiers and A/D converters, lowering the cost compared to CCD sensors [51]. 

To obtain depth images, the unit of active illumination emits modulated intensity light near infrared range. The light strikes the object or surface and it is reflected back to the camera. The reflected light is projected onto the image sensor used in the lens, CMOS in this case. The ToF camera sensor captures the reflected light and evaluates the distance information of every pixel. The phase difference is calculated from the relationship between four different values of electric charge. By correlating the signals transmitted and received, it is possible to calculate the distance from an illuminated object/scene by the sensor for each pixel [52]. The distance measurements D={d_i_ │ i = 1, … ,n} between image array and object are determined by Equation (2):

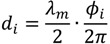
(2)
where *λ*_m_ is the wavelength of the modulation signal and *ϕ*_i_ is the pixel’s phase shift. 

The acquired color images and depth images in this first step are used to locate feature points of interest on the user’s body. Feature points of interest may include joints and locations corresponding to, for example, the patient’s left hand, left elbow, head, *etc.*

#### Beckon^TM^ SDK Architecture

**Figure 5:**
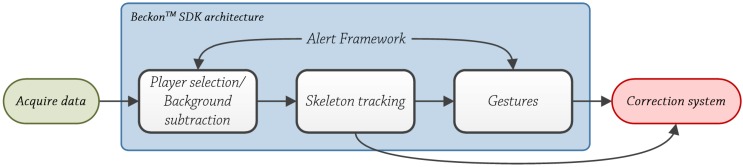
Beckon™ SDK architecture.

This module is carried out by the middleware provided by Omek Interactive. The Beckon™ SDK processes the raw depth map data along with color image data into intelligence about people’s positions or movements [53]. The individual steps shown in Figure 5: player selection/background subtraction, skeleton tracking and gestures were obtained from the Omek Beckon™ SDK 3.0 Windows Edition-Developer's Guide, included in the installation of the SDK. There are several methodologies and techniques in the literature to perform background subtracting based on ToF cameras [54,55] and based on structured light cameras [56,57]. 

To compute the skeleton, Moeslund *et al.* [58] identify two main categories of pose estimation algorithms based on human model:

- **Discriminative or model-free**. These methods don’t use models. In [59], Wren *et al.* use the bottom-up approach to track body parts, and in [60], Brand convert 2D sequences to 3D poses. There are two main groups: *example-based models* [61], which store a set of samples along with their corresponding pose descriptors; and *learning-based models* [62], which obtain the data from image observations using training samples.
- **Generative or model-based**. These methods employ a known model. In [63], Sigal *et al.* use a collection of loosely-connected body-parts using an undirected graphical model to track people. Merad *et al.* [64], use skeleton graphs to count people. There are two main groups [58]: *indirect models*, which use a model as a reference to analyze the data; and *direct models*, which use a 3D model of the human body along with kinematic data (direct or inverse) to analyze the obtained data**.**

With the skeleton data, the system is capable of identifying basic gestures like swipe or click. There are different approaches to detect (and learn) gestures. In [65], Miranda *et al*. use a combination of SVM system with decision forest to determine and learn the gesture. In this case, the input data is provided by a Kinect™ sensor. In [66], Boulic *et al*. use Inverse Kinematics (analytic and numeric) along with Kalman filters to estimate the end-effectors (human body parts) 3D position. The position is based on the previous one and the image data is obtained from a camera with a chroma key background. In [67], a complete review of several methods to recognize gestures is carried out.

#### Correction System

Once the skeletal model is created by Beckon™ SDK, the correction system starts to calculate the angles of the patient’s trunk, in this case the skeletal model.

**Figure 6:**
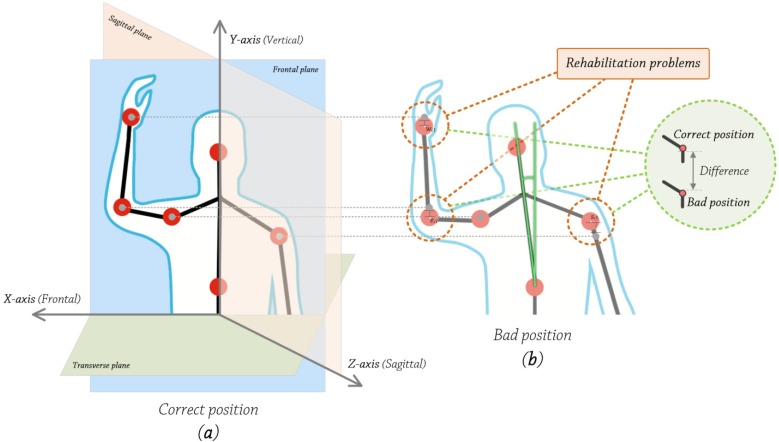
(**a**) Correct position *vs.* (**b**) Bad position.

Good posture is essential to health. As seen from Figure 6b, bad positions imply joint displacements, directly affecting the rehabilitation process. In fact, good posture guarantees that the blood circulates properly throughout the body. Poor posture results in energy loss due to muscle strain and because the air flow is restricted. Sitting in an awkward position for hours is bad for digestion, because the digestive organs are oppressed. Being accustomed to poor posture makes our muscles work more than necessary to maintain balance. The correct alignment of the spinal column is the key to the proper posture [68]. 

In rehabilitation, good posture of the spinal column and also the whole body is essential. There are several studies in the literature [69,70,71,72] which highlight the importance of good posture, and more specifically, the proper position of the spinal column.

With regard to techniques and methodologies which are currently applied to analyze the trunk’s positions, some are related with sports [73,74,75,76]: golf, baseball, *etc.*, activities in which the position is important. Up until a few years ago, mechanical systems and physical sensors were used to obtain information for a batch analysis, making it into a cumbersome and costly task. Since the emergence of Kinect™ in 2010, all the technology used has been made simpler, making this task more accessible both for professionals and people and more important, in real-time. Other are focused on post traumatic episodes such as strokes [77,78,79,80], to measure the position of patients.

The correction system only evaluates joints above the pelvis, because all of the patients who have taken part in the pilot test use a wheelchair. Beckon™ framework allows selecting individual joints, so the system does not track unnecessary data and the analysis is more efficient and faster. Human movements are described in 3D based on three planes and three axes. The human body moves along three main planes [81], see Figure 6a:

- **The sagittal plane** is a vertical plane which passes from the front of the body to the back, dividing it into two halves, left and right.
- **The frontal plane** is a vertical plane that passes from the one side end of the body to the other, dividing the body into two halves, anterior and posterior.
- **The transverse plane** is a horizontal plane which divides the body horizontally into upper and lower halves.

Human body rotates along three main axes [81]; see Figure 6a:

- **Frontal axis (X-axis)**. It runs from left to right and it is perpendicular to the vertical axis.
- **Vertical axis (Y-axis)**. In standing posture, it is positioned perpendicular to the supporting surface.
- **Sagittal axis (Z-axis)**. It runs from the rear surface of the body to the front surface, and it is perpendicular to X and Y axes.

**Figure 7:**
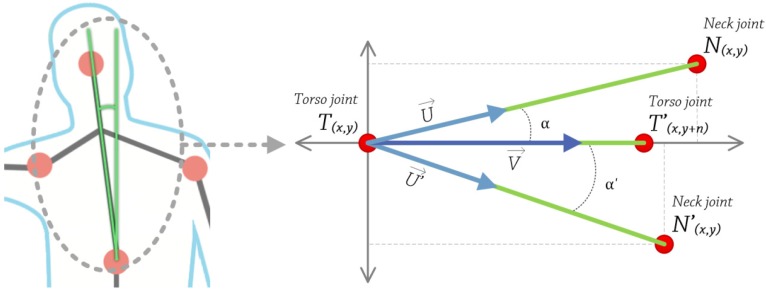
Trunk’s angle calculation.

The developed system evaluates trunk’s position in the frontal plane, the upper part specifically, and vertical axis, so movements like leaning forward or leaning backwards are not taken into account (see Figure 7). 

To calculate the trunk’s angle, we apply the following steps:

1. Obtain 2D coordinates, in pixels, of torso and some pixels of the upper the torso. Using these two points, construct a vector *V* that passes through them. This is calculated at the very beginning, and then used as a reference value.
2. Get 2D coordinates, in pixels, of neck and torso joints. Using these two points, construct a vector *U* that passes through them.
3. Calculate the angle between these two vectors *U* and *V*, using the following equation, Equation (3):

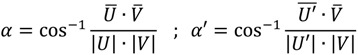
(3)

Steps number 2 and 3 were executed in every 5th frame (correction system calculations lasted between 2 and 4 frames, 0.12–0.24 s) captured by the camera, so the analysis is carried out three times every second (camera frame rate was approximately 15). During the test, the correction system causes the CPU load on the testing machines (system configuration: Intel^®^ Pentium^®^ D 805 @ 2.66 GHz, 2 GB of RAM, Nvidia^®^ GeForce^®^ 7300 LE, Microsoft^®^ Windows^®^ 7 Professional) to be between 50% and 60% and between 75 MB and 100 MB of RAM. The web application, during the recording, causes the CPU load on the testing machines to be between 20% and 30% and between 50 MB and 60 MB of RAM.

If α≠0º, the patient’s trunk is not vertically aligned. Due to the resolution of the camera (160 × 120 pixels), we established a threshold of 8 degrees according to the physiotherapists and based on alpha tests. The value can be modified through a configuration file, along with other parameters like camera resolution, frames per second, *etc.* This configuration capacity is vital, because every patient has a different amplitude of movement, so it is important to adjust the system. An 8° threshold value helps prevent false positives, so the system does not continuously notify for bad positions, which would affect the rehabilitation itself. If the angle is greater than 8° or greater than the configured value, the system will warn the user, indicating where to move, left or right and how much he/she should move, a little or a lot. This procedure is repeated during the exercise duration (five min on average).

## 6. Results

### 6.1. Experiment Example

**Figure 8:**
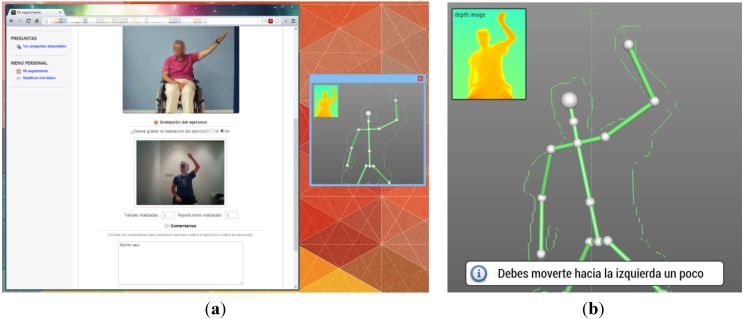
(**a**) Rehabilitation tool; (**b**) Warning message.

Below are some screenshots of the proposed system used in the pilot test: Carrying out an exercise through the web application, Figure 8a; Warning message for bad position indicating the patient to move left, Figure 8b.

### 6.2. Assessment Analysis

Both were 40 point questionnaires, and then were multiplied by 2.5 to get a value out of 100, in order to see the values easily. Questionnaire no. 1 is an internal generic questionnaire which was developed to evaluate the benefits of the systems, in terms of health. Our goal is to use this questionnaire in our systems and in as many others as possible, to convert it into a standard for the community. The scale values go from 1 to 4, avoiding middle-range values. Questionnaire no. 2 is SUS based. In SUS questionnaires, there are answers which subtract one from the user response and there are answers which subtract the user responses from 5, so all the scale values go from 0 to 4 [39]. 

As the two were 40 point questionnaires, we were able to compare them and obtain a relationship between them, as will be seen below when analyzing the data.

**Figure 9:**
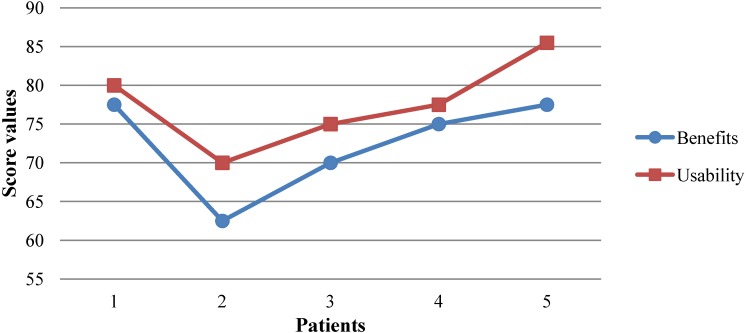
Results of questionnaires no. 1 and no. 2.

With respect to questionnaire no. 1, benefits to patients, patients (*n* = 5) obtained a median score (MS) of 72.5 with standard deviation (SD) of 6.846 and standard error of the mean (SEM) of 3.062 (α = 0.05). There was only one questionnaire below 70 points (20%) and 4 questionnaires were over 70 points (80%). Maximum value was 77.5 and minimum value was 62.5. This is reflected in Figure 9. Patients (*n* = 5) obtained a MS = 77, SD = 4.809 and SEM = 2.151 (α = 0.05) in the questionnaire no. 2, usability of the system. In this case, our system obtained the 3rd quartile, meaning that our system was considered acceptable with a value of 77, grade C [82]. This is reflected in Figure 9. There was only one questionnaire below 75 points (20%) and there were four questionnaires over 75 points (80%). Maximum value was 85.5 and minimum value was 70. This is reflected in Figure 9.

**Figure 10:**
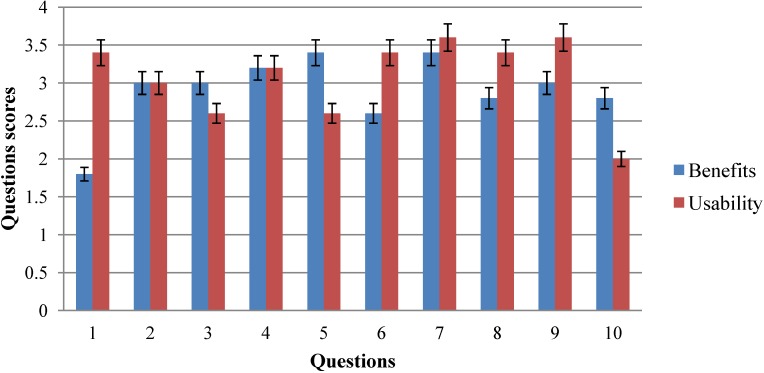
Questions values of questionnaire no. 1 and no. 2.

With respect to responses, patients obtained a MS = 2.9000, SD = 0.274 and SEM = 0.122 (α = 0.05) in questionnaire no. 1. The question with highest score was no. 5, I think that I would like to use the system frequently, because it helps me to improve my quality of life and no. 7, After using the system, I think I am more independent (dressing, toileting, etc.), both with an average value of 3.4, and the answer with the lowest score was number 1: Before using the tool, I think that my health problems were worse than other people in the same situation, with an average value of 1.7. Nine questions received scores of over two points (90%), and three questions were over three points (30%). This is reflected in Figure 10. 

As for questions of questionnaire no. 2, patients obtained a MS = 3.08, SD = 0.129 and SEM = 0.086 (α = 0.05). The answer with the highest score was no. 7, I was able to read every option of the system, and no. 9: I found the various functions in this system to be well integrated, both with an average value of 3.6. The question with lowest score was no. 10: I needed to learn a lot of things before I could get going with this system, with an average value of 2. Nine questions out of ten received scores of over two points (90%), and six questions were over three points (60%). This is shown in Figure 10. The correlation between usability and benefits based on questionnaires was 0.968. This correlation is significant because p < 0.05 Specifically: t(5) = 6.746, p = 0.006 (p < 0.05).

## 7. Discussion

Any person with MS, regardless of the severity of their disability, needs regular physical activity [83,84,85]. Lack of physical activity could have dramatic consequences for their health. The exercises not only provide a sense of well-being but are also important in preventing other problems associated with MS. Published studies show that rehabilitation therapy in patients with MS improves their disability and quality of life. Since the effects decline over time, it will be essential to regularly monitor patients [86]. Few authors have developed a specific technique for the treatment of MS patients. The National Multiple Sclerosis Centre (NMSC) in Belgium works in this field, where Physiotherapy is integrated into a 24-hour action plan. Applied therapy sessions are organized in units that include an exercise program, muscle stretching and perineal reeducation, individually or in groups [87]. Together with Physiotherapy and Rehabilitation, Occupational Therapy is very important [88]. Many patients with MS, in varying degrees, have trouble performing daily activities due to fatigue, tremors, spasticity, *etc.* In this sense, the objective of occupational therapy is to achieve maximum independence in daily activities, teaching, giving advice and making functional adaptations according to each individual.

The tracking system we developed offers a new information channel for professionals and patients to improve the monitoring of the physical aspect of the pathology, MS in this case. Through the tracking system, patients see their position during the exercise in real time, helping them (or the care givers in case of dependency) to perform the exercise correctly, because poorly performed exercises could aggravate their muscle imbalances and worsen their health. The technology used, time-of-flight, is new in this kind of systems, and until now has only been used in assembly lines, bioengineering, medicine and videogames [89,90,91,92,93]. This technology was very expensive, more than $2,500, until recent years, but there now are several devices available on the market at low prices. Examples include Softkinetic™ solutions with DS311 ($299) and DS325 ($249) cameras and Microsoft™, with the next version of Kinect™ for Xbox™ One ($499 in a pack) and PC-Windows ($399) (Xbox™ One version would be released on 22 November 2013, and the PC-Windows version is scheduled to be on the market the first to second quarter of 2014). In this way, our system can reach more users and can used not only at medical centers, but even in patients’ homes, with the benefits this implies: social, because it is an improvement in their quality of life; and economic, because patient’s empowerment is enhanced and the available assets (people, materials and infrastructures) are used more effectively.

To determine this, we used two questionnaires, one to evaluate the benefits of the system we developed, and another one to evaluate its usability. The latter is one of the most highly recommended to validate usability, as seen in the literature. Preliminary results are very promising, in spite of the limited scale (*n* = 5). The first questionnaire scored a total of 72.5 points over 100, which indicates it has great benefits for patients. The second questionnaire scored a total of 77 points over 100, with 68 or more points considered to be a valid system [39]. The correlation coefficient between usability and benefits showed a value *r* = 0.968 (*p* = 0.006), so the results were consistent, positive considerable correlation, and both tests were reliable. High usability implies high benefits.

## 8. Conclusions

The proposed system offers a new way for professionals to continue with personalized therapy for patients outside the medical center premises. Professionals have the opportunity to combine traditional therapies with online therapies, having effective control over the evolution of the patients. For their part, patients have two systems that complement each other: traditional therapies and online therapies. One day per week patients go to the medical center and have traditional therapy, and the rest of the week, they perform the exercises assigned by the professional at home.

The tracking system developed, along with the web application, forms an integral rehabilitation system. This integral tool offers real time feedback, the tracking system, and 24/7 access to the exercises and rehabilitation, the web application. In addition to this, the system can be used both at the medical center and at the patient’s home, so the patient may choose the preferred option. This working mode has some benefits. On the one hand, patients are in contact with professionals, which is very important; and on the other hand, patients continue their rehabilitation at home, involving their family members. This helps to empower patients because they are the ones who manage their disease and evolve in the best way asking for more treatment, feedback, *etc.* In short, patients play the leading role.

Preliminary results, based on a limit number of patients (*n* = 5) are promising. Both, usability and benefits of the system have achieved good numbers, 77% and 72.5% respectively, indicating that the path taken is correct and future research should continue with the same methodology.
